# Mouse Models to Study Dengue Virus Immunology and Pathogenesis

**DOI:** 10.3389/fimmu.2014.00151

**Published:** 2014-04-10

**Authors:** Raphaël M. Zellweger, Sujan Shresta

**Affiliations:** ^1^Division of Vaccine Discovery, La Jolla Institute for Allergy and Immunology, La Jolla, CA, USA

**Keywords:** dengue, vaccines, mouse models, antibody-dependent enhancement, adaptive immunity

## Abstract

The development of a compelling murine model of dengue virus (DENV) infection has been challenging, because DENV clinical isolates do not readily replicate or cause pathology in immunocompetent mice. However, research using immunocompromised mice and/or mouse-adapted viruses allows investigation of questions that may be impossible to address in human studies. In this review, we discuss the potential strengths and limitations of existing mouse models of dengue disease. Human studies are descriptive by nature; moreover, the strain, time, and sequence of infection are often unknown. In contrast, in mice, the conditions of infection are well defined and a large number of experimental parameters can be varied at will. Therefore, mouse models offer an opportunity to experimentally test hypotheses that are based on epidemiological observations. In particular, gain-of-function or loss-of-function models can be established to assess how different components of the immune system (either alone or in combination) contribute to protection or pathogenesis during secondary infections or after vaccination. In addition, mouse models have been used for pre-clinical testing of anti-viral drugs or for vaccine development studies. Conclusions based on mouse experiments must be extrapolated to DENV-infection in humans with caution due to the inherent limitations of animal models. However, research in mouse models is a useful complement to *in vitro* and epidemiological data, and may delineate new areas that deserve attention during future human studies.

## Introduction

Dengue virus (DENV) is the etiologic agent of the self-limited febrile illness dengue fever (DF), as well as the potentially lethal severe dengue disease (previously referred to as dengue hemorrhagic fever and dengue shock syndrome, DHF/DSS). Infection with DENV is often asymptomatic in humans (1). Symptomatic infections are characterized by: fever, retro-orbital headache, muscle, joint and bone pain, nausea, vomiting, abdominal pain, mucosal bleeding, and low platelet count. In the most severe form of the disease, severe bleeding, organ dysfunction, vascular permeability, and shock can occur (2, 3).

The four serotypes of DENV are the most prevalent mosquito-borne viruses that affect humans (4). In tropical and sub-tropical regions, it is estimated that 2.5–3.6 billion people are at risk of infection in over 100 countries (2, 5, 6). The development of an adequate mouse model for dengue infection has been challenging, mainly because DENV clinical isolates fail to replicate or cause pathology in wildtype (WT) mice. A reliable mouse model of DENV-infection would be an excellent complement to *in vitro* data and epidemiological studies, allowing for testing of hypotheses based on human observations, and providing insights into mechanisms of pathogenesis and immunity to DENV.

## Existing Animal Models of DENV-Infection

Developing a relevant animal model for DENV-infection has been a long-standing challenge. The lack of an adequate animal model for DENV-infection is often mentioned as a major hurdle to a better understanding of DENV pathogenesis in humans. Numerous efforts to overcome this difficulty have resulted in many animal models. Though not perfect, these models have been useful to complement *in vitro* and human studies.

### Non-human primate models of DENV-infection

Non-human primate (NHP) models of DENV-infection exist, and have been recently reviewed (7). Viremia and humoral immune responses can be detected in NHP after DENV-infection, but usually no clinical signs of disease are observed (8–12). One study reported hemorrhage after infection with DENV (13). Due to the absence of signs of disease, it is difficult to study DENV-induced pathology in NHP, but NHP can be used for testing immunogenicity, safety, and/or protective efficacy of dengue vaccine candidates by measuring induction of anti-DENV antibodies and magnitude and duration of viremia after vaccination or challenge (14–23). Thus, in the context of vaccination, the antibody and viremia data from NHP models have been used to provide correlates of protection from infection but not from pathogenesis.

### Wildtype mouse models of DENV-infection

In many WT mouse models, intravenous (i.v.) or intraperitoneal (i.p.) DENV-infection results in neurological abnormalities but not the usual clinical signs observed in humans (24–27). Involvement of the central nervous system during DENV-infection in humans has generally been considered unusual (28, 29), although recent studies [reviewed in Ref. (3)] suggest that it is a frequent complication (30–33). Central nervous system involvement is now considered criteria for severe dengue in the World Health Organization (WHO) case classification (2, 3). Intracranial (i.c.) inoculation of DENV in WT mice also results in neurological symptoms and death (34–37), however this inoculation route does not accurately mimic natural infection, which occurs when DENV-infected mosquitoes feed on a human. In C57BL/6 mice, a high inoculum of the non-mouse-adapted DENV2 strain 16681 resulted in systemic hemorrhage after intradermal (i.d.) infection (38) and abnormal liver function after i.v. infection (39). It is still a matter of debate which experimental route of infection (i.d. or i.v.) is the most relevant. While neither perfectly mimics the bite of an infected mosquito, i.d. and i.v. routes of infection are often used in laboratories when mosquito-mediated infection may not be possible. In another immunocompetent mouse model, presence of DENV was transiently detected in the serum of ICR, ddY, and Balb/c mice after i.p. injection of DENV-infected K562 cells (40). Collectively, the absence of overt signs of disease in WT mice after DENV-infection has been a major drawback of WT models to date.

### Mice deficient in IFN-α/β and -γ receptors (AG129) to study DENV-infection

In humans, DENV inhibits IFN signaling to establish infection, whereas DENV is unable to do so in mouse cells (41–43). Specifically, DENV NS5 can bind to and degrade human STAT2 (44), but not mouse STAT2 (41) and the DENV NS2B3 proteolytic complex can degrade human STING but not its mouse homologue (42, 43). The extreme sensitivity of DENV to type I IFN is highlighted by a recent study demonstrating effective viral clearance in mice deficient in both IRF-3 and IRF-7, which mount a weak and delayed type I IFN response during DENV-infection (45). Another study has demonstrated in mice that type II IFN efficiently limits DENV spread (46). Taken together, the high sensitivity of DENV to IFN and the fact that DENV inhibits IFN signaling in humans but not in mice could possibly explain why DENV clinical isolates do not easily replicate in WT mice.

In contrast to WT mice, mice deficient in IFN-α/β and -γ receptors on 129/Sv genetic background (AG129) support robust levels of DENV replication (47), and have thus widely been used as a mouse model of DENV-infection. Intraperitoneal infection with a mouse-adapted DENV-strain was lethal in AG129 mice, regardless of their age (47). While i.p. may not accurately mimic the natural route of infection, similar results were obtained in subsequent studies using i.v. inoculation. In those studies, i.v. infection of AG129 mice with a mouse-adapted DENV serotype 2 strain (DENV2) resulted in non-paralytic lethal dengue disease with signs of vascular permeability and TNF release (48, 49). This model has been used to demonstrate that passive transfer of sub-protective levels of anti-DENV antibodies before infection can turn a mild illness into a lethal disease recapitulating many signs of severe dengue disease, including viremia, cytokine storm, low platelet counts, elevated hematocrit, increased vascular permeability, and intestinal hemorrhage (50, 51). AG129 mice were also used to demonstrate the therapeutic efficacy of anti-DENV antibodies lacking the ability to bind the Fcγ receptor (52). Recently, i.p. infection with a non-mouse-adapted DENV2 strain (D2Y98P) was shown to cause cytokine storm, organ damage, and vascular leakage in AG129 mice (53, 54). If infected with lower doses of DENV2, AG129 mice do not develop severe dengue-like lethal disease, but instead neurological symptoms appear and result in paralysis later on (46, 51).

### Mice deficient in IFN-α/β receptors (IFNAR^−/−^) to study DENV-infection

In contrast to AG129 mice lacking both IFN-α/β and -γ receptors, mice lacking IFN-α/β receptor only (IFNAR^−/−^) do not develop paralysis after i.v. infection with DENV2 (mouse-adapted) because IFN-γ and CD8^+^ T cells prevent DENV-induced pathology in the central nervous system (46, 55). Instead of paralysis, these mice develop a severe dengue-like disease when infected with sufficiently high DENV2 challenge doses (46) or in the presence of sub-neutralizing (and enhancing) anti-DENV antibodies (51). IFNAR^−/−^ mice have also been used to demonstrate that both CD4^+^ and CD8^+^ T cells play a protective role in the context of primary DENV2 infection (mouse-adapted strain, i.v. infection) and peptide vaccination (56, 57).

Recently, IFNAR^−/−^ mice have been crossed with transgenic mice expressing human HLA molecules in order study anti-DENV-T cell responses restricted by human HLA (58). Observations made in those mice confirmed the role of NS3 and NS5 as major targets of the T cell response in humans (59), identified several novel human DENV-T cell epitopes, and highlighted the dominance of HLA-B*0702 restricted responses (58).

Sustained DENV replication and severe dengue disease manifestations in IFNAR^−/−^ and AG129 mice are a clear advantage to study DENV pathogenesis *in vivo*, but the absence of intact IFN signaling is a limitation that must be taken into account when interpreting data. In particular, immune responses to DENV are altered in IFNAR^−/−^ and AG129 mice compared to WT mice, as IFN is a key component of the immune system. Therefore, findings made in IFNAR^−/−^ and AG129 mice may not accurately reflect what would happen in a fully immunocompetent environment. It would be desirable to validate the findings made in IFN-deficient mice with another model, or with data from epidemiological studies.

### “Humanized” mice to study DENV-infection

Another approach aimed at modeling DENV-infection in mice has been to graft human tumor cells into severe combined immunodeficient (SCID) mice lacking T and B cells. As SCID mice are unable to reject the graft, the transplanted human tumor cells provide a permissive environment for DENV replication. DENV replication was observed after transplantation of human hepatocarcinoma hepG2 (60) or human leukemia K562 cells (61). Viremia and some signs of disease (mild hemorrhage, thrombocytopenia, and elevated TNF levels) were detected in one study (60), but ultimately mice succumbed to paralysis. As DENV replication is restricted to the transplanted transformed human cells, extrapolation of results to human disease may be difficult.

Human CD34^+^ hematopoietic stem cells have also been used to reconstitute irradiated NOD/SCID (62) or NOD/SCID/IL-2Rγ-null (63–65) mice. Graft-rejection is minimized in NOD/SCID/IL-2Rγ-null mice due to dramatic defects in both adaptive and innate immune systems. Therefore in irradiated NOD/SCID/IL-2Rγ-null mice, the efficiency of reconstitution by human CD34^+^ cells is maximized. In the resulting “humanized” chimeras (hu-NSG), human cells of the adaptive and innate immune systems develop with various degrees of reconstitution depending on the animal, the organ, and the laboratory (62, 66, 67). Fever, rash, viremia, erythema, and thrombocytopenia have been observed in humanized mice after DENV-infection (62, 63, 65), and human cells were infected in the bone marrow, spleen, and blood (62). Production of anti-DENV IgM and cytokines was also observed in those mice (64, 65, 68). Similar to NOD/SCID/IL-2Rγ-null mice, immunodeficient RAG2^−/−^/γc^−/−^mice can also be reconstituted with human hematopoietic stem cells (67). DENV-infection of humanized RAG2^−/−^/γc^−/−^ resulted in viremia, fever, and production of human IgM and IgG (69). Recently, NOD/SCID/IL-2Rγ-null mice have been co-transplanted with cord blood hematopoietic stem cells and human fetal thymus and liver tissues, which resulted in higher immune responses to DENV-infection (neutralizing IgM and IFN-γ production) (70). In another study, NOD/SCID mice were grafted with human fetal liver and thymus prior to reconstitution with human CD34^+^ hematopoietic stem cells (71). In those mice, infection with DENV resulted in viremia, infection of human leukocytes, cytokine production, and production of DENV-specific IgM. Also, viral RNA levels were reduced by administration of an adenosine nucleoside inhibitor of DENV, either simultaneously or after infection (71). Based on those results, the authors suggest that “humanized” mice could be used to test the efficacy of anti-viral drugs or, potentially, the protective efficacy of vaccine candidates.

While using mice reconstituted with human cells offers the possibility to study DENV-infection of human cells *in vivo*, this approach is labor-intensive and important variations are observed in the degree of reconstitution of the recipients (62, 66, 67). In addition, cellular and molecular interactions between the grafted human cells and the murine environment may be absent or altered compared to the interactions that would take place in a natural environment. Therefore, transferred human cells may not function in the murine environment as they would in their natural (human) environment. Overall, few signs of severe dengue disease are observed in “humanized” mice, and some aspects of both adaptive and innate responses are not fully functional (67). Cellular immune responses are usually observed after viral infection, but isotype class-switch does not readily occur in these mice (67).

Various mouse models of DENV-infection exist, each one with specific strengths and limitations. While improvement of the current models would be desirable, these models have been used to test anti-viral drugs and vaccine candidates, or to provide insights into mechanisms of DENV pathogenesis. As pathogenesis and immune responses after DENV-infection probably differ between mice and humans, extrapolation of findings made in mice to DENV-infection in human should be done with caution. This limitation has to be kept in mind when reviewing any data generated in animal models.

## Use of Mouse Models for Drug and Vaccine Development

Efforts to develop efficient therapeutics and vaccines against DENV are still ongoing. Studies using mouse models of DENV-infection could be used for drug development, or provide valuable information on safety, immunogenicity, and efficacy of vaccine candidates before they reach the clinical testing stage.

### Anti-viral testing in mouse models of DENV-infection

Several anti-viral drug candidates have been tested in WT mice after i.c. infection, or in AG129 mice. The alpha-glucosidase inhibitor castanospermine (a natural alkaloid) can inhibit DENV-infection by preventing correct folding of viral structural proteins (72) and prevent mortality after i.c. DENV challenge in WT mice (73). Contrary to WT mice, in which DENV is lethal only after i.c. challenge, AG129 mice support DENV replication and show signs of severe dengue disease following i.p. or i.v. infection with certain DENV-strains (48–54). Therefore, this model has been widely used to test the ability of anti-viral drugs to limit DENV replication and dissemination, and/or prevent signs of disease. Mortality, viral load, and signs of disease can all be used as readouts for the drug efficacy. Iminosugars have anti-viral properties, as they prevent correct protein folding by inhibiting glycoprotein processing enzymes α-glucosidases I and II (74). In AG129 mice, various iminosugars have been shown to reduce viremia, prevent cytokine storm, and/or limit mortality after DENV-infection (75–80). One study showed that co-administration of ribavirin enhanced the anti-viral activity of the iminosugar (76). Furthermore, an adenosine nucleoside could limit viremia and reduce mortality in DENV-infected AG129 mice, presumably by blocking viral RNA synthesis (81, 82). Another compound, which inhibited RNA translation, reduced peak viremia in AG129 mice (83). Other DENV inhibitors targeting the NS3 helicase (84) or the capsid protein (85) have also successfully reduced viremia and organ viral titers in AG129 mice. In recent years, AG129 mice have increasingly become the standard mouse model for *in vivo* testing of anti-viral candidates that impact viral replication through type I and II IFN-independent pathways.

### Mouse models to test safety of DENV-vaccine candidates

A successful vaccine should induce protective immunity in the host without causing pathology. This is particularly true for live-attenuated vaccines, which are often derived from more virulent parental strains. Mouse models in which DENV can replicate and cause disease can be used to assess the safety of vaccine candidates. Duration and magnitude of viremia, morbidity, and (potentially) mortality after vaccination can be used as readouts to assess the degree of attenuation of vaccine candidates compared to the parental, non-attenuated strain. The degree of attenuation of live DENV-vaccine candidates has been tested in suckling mouse brains (86) and in SCID mice transplanted with human liver cells (18, 87, 88). The WHO has suggested that the AG129 mouse model may be adequate to test the safety of live-attenuated DENV-vaccine candidates, as the potential to cause disease (compared to the parental, non-attenuated strain) can be evaluated in those mice (89). However, the authors point out the difficulty of interpreting the results, as AG129 mice lack both type I and II IFN receptors. In addition, the risk of inducing antibody-dependent enhancement after vaccination can readily be assessed in AG129 mice, as viral titers and morbidity have been shown to increase when sub-neutralizing amounts of anti-DENV antibodies are administered prior to infection (50–52, 90).

### Mouse models to test immunogenicity and efficacy of DENV-vaccine candidates

Immunogenicity and efficacy of DENV-vaccine candidates have also been tested in both AG129 and WT mice. The effect of immunization on subsequent challenge can easily be observed in AG129 mice, as the appropriate DENV-strain will replicate to measurable levels and cause pathology in those mice. A protective vaccine should reduce viral titers and pathology, as well as increase mean survival time after lethal challenge. Live-attenuated monovalent and tetravalent vaccine candidates induced neutralizing antibodies in AG129 mice, and protected from lethal DENV challenge (23, 91, 92). One study reported induction of DENV-specific CD8^+^ T cells after vaccination of IFNAR^−/−^ mice (23). Recently, immunization with a non-propagating alphavirus replicon particle expressing a truncated version of the DENV envelope protein induced both cellular and humoral immunity, and was able to protect AG129 mice form lethal i.v. DENV challenge (90). The limitation of using AG129 mice is that vaccine-induced responses may be altered relative to immunocompetent mice or humans, as both type I and II IFN may regulate vaccine-induced immune responses.

Immune responses after vaccination with non-replicating DENV-vaccine candidates have also been investigated in WT mice, and neutralizing antibody responses were elicited after immunization with subunit vaccines containing portions of the DENV envelope protein (20, 93, 94) or after DNA priming and protein boosting with various combination of envelope and non-structural proteins (95, 96). Many studies used i.c. lethal DENV challenge to test the protective efficacy of DENV-vaccine candidates in WT mice (20, 34, 35, 37, 97–99). In those studies, the readout for protection was reduction of morbidity and/or increased survival after i.c. lethal challenge.

In general, testing the efficacy of DENV-vaccination in WT mice is difficult, as DENV challenge will not result in measurable viral titers or pathology in WT mice, with the exception of viral replication in the central nervous system after i.c. challenge (as mentioned above). One way to circumvent this problem is to use an adoptive transfer system: since many vaccines are immunogenic in WT mice, WT animals can be vaccinated and, subsequently, T cells, B cells, or serum can be transferred (alone or in combination) into naïve AG129 recipients prior to challenge. The protective or potentially enhancing effect of immune T cells, B cells, or serum can be assessed by monitoring changes in viral load or pathology in the AG129 recipient, where DENV readily replicates and causes disease. Thus, in this adoptive transfer system, the AG129 mice serve as a highly stringent challenge assay. This approach has been recently used to assess the relative contribution of T cells and antibodies after vaccination, revealing that not only antibodies, but also T cells could efficiently contribute to protection after vaccination (90).

## Mouse Studies to Complement Human Studies

*In vitro* experiments cannot fully reflect the complexity of the whole organism, and human studies are descriptive by nature. In human studies, key parameters such as viral strain, dose, sequence of infection, and/or interval between infections are often unclear or cannot be easily manipulated. Therefore, research in mouse models is an ideal complement as it uses whole organisms, and allows for variation of many experimental parameters. Care must be taken while extrapolating conclusions based on experiments carried out in mice to dengue disease in humans, as there is some artificial component in all the models described so far.

### Mouse models to confirm observations made in human studies

Research in mouse models can be useful to verify hypotheses that have been based on epidemiological observations, or to gain insights into the mechanistic aspects of phenomena that have been observed in humans. For example, the hypothesis that antibody from a previous infection with a heterotypic virus (or acquired from an immune mother) can increase severity of disease upon re-infection (antibody-dependent enhancement, ADE) has been postulated a long time ago (100, 101). Increased infection of target cells in the presence of sub-neutralizing amounts of antibody is readily observed *in vitro* (102–106). In monkeys, viremia increases after transfer of antibodies, but no signs of disease are observed (107, 108). Recently, the AG129 mouse model was used to demonstrate that passive transfer of sub-neutralizing amounts of anti-DENV antibodies could turn a mild illness into a lethal disease upon DENV-infection (50, 51). The antibody-induced severe dengue disease displayed many signs observed during severe dengue disease in humans: elevated viral RNA titers in multiple organs, cytokine storm, low platelet counts, elevated hematocrit, increased vascular permeability, intestinal hemorrhage, and ultimately death. Those studies also formally confirmed the involvement of the Fcγ-receptor during ADE *in vivo*, and a subsequent study demonstrated the therapeutic potential of anti-DENV antibodies that no longer bind to the Fc-γ receptor (52).

Another example is the association between high viremia and disease severity. In humans, viremia is higher and persists longer during severe dengue compared to DF (109–113). This has also been observed in mice, where disease severity has been shown to correlate with higher viremia. Mouse models have been used to explore various experimental scenarios in which viral or host factors influence virus levels and, consequently, severity of disease. In the single-deficient IFNAR^−/−^ mice, severity of disease correlated with the amount of virus inoculated i.v. (46). The same study showed that, at equal viral doses, the presence of intact IFN-γ signaling reduced systemic viral spread and severity of disease. Other mouse model studies examining mechanisms of anti-viral innate immunity revealed an essential role for MAVS (55) and IRF-3/7 (45) in the initial induction of type I IFN response and control of viral replication, and for STAT1 and STAT2 in the late induction of type I IFN response and control of viral replication (114). In various studies using IFN-α/β and -γ receptor deficient mice, the presence of sub-neutralizing levels of antibodies increased viremia and resulted in severe dengue-like disease (50–52). CD8-depletion before infection resulted in increased viral loads in another murine study (56). Many studies have also shown that virulence factors influence the outcome of the infection. One study showed that two mutations in the envelope protein of DENV were sufficient to delay virus clearance from serum, increase systemic viral loads, and induce severe dengue disease in mice (49). Similarly, a single amino acid change in the non-structural protein NS4B of a non-virulent DENV-strain caused increased RNA synthesis, increased viremia, and decreased survival time in AG129 mice (54). Therefore, while the observation that higher viremia correlates with disease severity has been widely made in humans, studies in mice have confirmed and extended those observations by demonstrating how particular host- and virus-associated factors impact viremia and disease severity; those factors include specific elements of innate and adaptive immune responses, presence of sub-neutralizing antibodies, size of the initial virus inoculum, and virulence of the infecting viral strain.

### Mouse models to explore the role of different components of the immune system

Human studies have suggested that the immune system can be involved in both protection and immunopathology during DENV-infection. For example, studies have shown that passively transferred anti-DENV maternal antibodies can initially protect infants against disease (115–118), but later on, as titers decrease, sub-protective levels of antibody favor the development of severe disease (115, 116, 118). T cells have also been suspected to increase disease severity: cross-reactive T cells raised during a primary infection have been suggested to dominate the response during a secondary, heterotypic infection, resulting in delayed viral clearance (119), altered cytokine production profile (120), or excessive cytokine release (121). However, another study showed that while cross-reactive T cells did dominate the response during secondary heterotypic infections, they did not show any sign of impairment (122). On the contrary, results of this study supported an HLA-linked protective role for CD8^+^ T cells.

Based on these observations, it is clear that a better understanding of the relative contribution of the cellular and the humoral arms of the immune system to protection and/or immunopathogenesis during DENV-infection is needed. Tractable, genetically manipulable mouse models enable the investigation of the role of different components of the immune system during DENV-infection, re-infection, or after vaccination. Passive transfer of serum, adoptive transfer of various T cell subsets, or depletion experiments should allow for a better understanding of the protective or potentially pathogenic role of the different components of the immune system, either alone or in combination. Passive transfer of anti-DENV antibodies can protect mice from intracerebral lethal challenge (123) or increase mean survival time after i.v. challenge (51). However, sub-neutralizing levels of antibodies can also increase the level of infection and/or the severity of disease in mice (50, 51). One study showed that the same neutralizing antibody could protect or enhance infection, depending on the amount transferred prior to infection (51). This is an *in vivo* confirmation of the observation made *in vitro* that even antibodies that are neutralizing can enhance infection when used at sub-neutralizing concentrations (103). A protective role for T cells during DENV primary infection was established in mice by showing that CD8-depletion prior to infection increased viral load in various organs upon infection (56). Similarly, vaccination with CD8-epitopes (56) or CD4-epitopes (57) reduced viral load upon DENV-infection, showing that priming cellular responses before challenge was beneficial for the host. Passive transfer of homotypic or heterotypic serum or adoptive transfer of homotypic or heterotypic splenocytes before DENV challenge reduced viral load in various organs (124). Similarly, a protective role for both cross-reactive T cells and B cells was shown in AG129 mice (125). Recently, the relative contribution of the humoral and the cellular arms of the immune system were assessed in mice after vaccination with a protective DENV-vaccine candidate (90). After vaccination, short-term protection was mediated by CD8^+^ T cells. Later on, CD8^+^ T cells were or were not required for protection, depending on the immunization schedule. All those studies explored the role of various components of the immune system in isolation from the other ones, which would be difficult in human studies.

Despite sustained vaccine development efforts, there is still no clinically approved vaccine against DENV (126–133). In terms of dengue vaccine development efforts to date, measuring neutralizing antibodies by plaque reduction neutralization test (PRNT) may not accurately predict the protective efficacy of a vaccine against DENV (133–135). Therefore, a better understanding of the relative contribution of the different components of the immune system to protection and/or pathogenesis is crucial to develop better correlates of protection as well as a safe and effective DENV vaccine.

### Mouse models to suggest areas of interest for future human studies

While research in mouse models has confirmed some of the hypotheses based on human studies, some of the findings made using mouse models need validation with human data. In this respect, the value of the mouse model is to point out possible areas of importance for future human studies. For example, elevated liver DENV titers and infection of liver sinusoidal endothelial cells (LSECs) were observed during antibody-induced severe dengue disease in mice (51). In humans, liver pathology is often observed after DENV-infection, and the degree of dysfunction may correlate with disease severity (136–140). In addition, DENV RNA or antigen has been found in the liver of patients who succumbed to DHF/DSS (141–143). Recently, a case of DENV transmission following a liver transplant has been reported (144). However, the cellular localization of DENV replication in the liver is still controversial, and possibly deserves more attention in future human studies.

Similarly, a protective role for T cells during DENV-infection has been widely shown in mice (56, 57, 90), but human studies have only recently started to explore more in depth the protective role of T cells during DENV-infection (122). Studies in mice could suggest which T cell subsets are required to limit viral replication, and which T cell-mediated mechanisms protect against dengue pathogenesis during both primary and secondary infections, as well as explore further the role of sero-specific and cross-reactive T cells in protection and/or pathogenesis. IFNAR^−/−^ mice crossed to transgenic mice expressing human HLA molecules will also be valuable to explore anti-DENV-T cell responses restricted to human HLA. Subsequently, human studies will be necessary to verify if the findings made in mice apply to DENV-infection in humans.

## Conclusion

While the perfect mouse model of DENV-infection has yet to be established, existing murine models of DENV-infection have, within their own limitations, been invaluable tools to complement and/or expand observations made *in vitro* or in epidemiological studies. Each model has strengths and weaknesses that must be taken into consideration when assessing data. Therefore, the choice of the best-suited model to answer a particular question is of critical importance. Mouse models have been useful to test the safety and efficacy of vaccine candidates or potential anti-viral drugs, verify hypotheses based on human studies, gain insights into mechanistic aspects of DENV-induced immunity or pathology, and suggest areas worth attention in future human studies. As with any animal model, extrapolation of findings made in mice to DENV-infection in humans must be done with care. In the future, refinement and improvement of existing models may overcome some of the boundaries of today’s models, and expand the knowledge that murine models can generate about mechanisms of DENV pathogenesis and immunity.

## Conflict of Interest Statement

The authors declare that the research was conducted in the absence of any commercial or financial relationships that could be construed as a potential conflict of interest.
